# Chemistry of the Metals—Platinum

**Published:** 1854-04

**Authors:** Reginald N. Wright


AMERICAN JOURNAL

OF

DENTAL SCIENCE.

Vol. IV. NEW SERIES—APRIL, 1834. No. 3.

### ORIGINAL COMMUNICATIONS.

### ARTICLE I.

*Chemistry of the Metals—Platinum.* By Professor Reginald N. Wright, A. M., M. D.

(Continued from page 191.)

*Oxyds of Platinum.*—It is probable that only two real oxyds of this metal exist, since all our experiments only determine the *positive* existence of two. A hypothetical existence is, however, allowed to four, viz. *suboxyd, protoxyd, sesquioxyd and peroxyd.* Its affinity for *oxygen,* whether it is exposed to the air or to the action of perfectly pure gas, is so trifling, that we may safely say that they have no disposition to unite under *ordinary circumstances.*

If a powerful electric discharge is passed through platinum—in the shape of fine wire or leaf—a dark colored powder is produced, which used to be considered an oxyd of the metal, but it is very questionable, whether or not, it is the metal in a state of minute division.

*Suboxyd of Platinum* may be formed by exposing perchloride of the metal in aqueous solution, to the action of *nitrate of mercury*, also in solution, the result being a pulverulent mass, having a dark color, which, upon the application of heat, gives off the mild chloride of mercury (calomel) leaving behind a black mass, generally regarded as an oxyd.

*Protoxyde of Platinum.*—To produce this substance, take a solution of oxyd of potassium (caustic potassa) and heat in connection with it, *protochloride of platinum;* a black oxyd is formed, which is only in part thrown down, a portion being absorbed by the alkali used, but which may be precipitated admirably by means of sulphuric acid, which has been diluted. Reduction may now be accomplished by means of heat, attended with the escape of aqueous vapors and some oxygen gas. The addition of acids will accomplish slow solution, and we have, as the result, metal, and a higher state of oxydation.

The following account of the *sesquioxyd and peroxyd*, is copied from *Brande:*

*Sesquioxyd of Platinum.*—"When sulphate of platinum is decomposed by ammonia, and the precipitate boiled in a weak solution of potassa, and cautiously dried, it constitutes *fulminating platinum;* when this is digested in nitric acid, a gray powder remains, composed of 100 platinum, 11-86 oxygen. (*E. Davy, Phil. Trans.*, 1820.) When spongy platinum is heated to redness in an open vessel with caustic potassa, and the product, when cold, washed with water, a gray powder is obtained, which is partly dissolved by the alkali; the residue, washed with dilute nitric acid, and afterwards with water, is also supposed to be sesquioxyd of platinum, consisting of

| | | | | E. Davy. |
|---|---|---|---|---|
| Platinum, . . . . . . . | 1 | 99 | 89-2 | 89-5 |
| Oxygen, . . . . . . . | $1\frac{1}{2}$ | 12 | 10-8 | 10-5 |
| Sesquioxyd of platinum, | 1 | 111 | 100-0 | 100-0 |

*Peroxyd of Platinum.*—When sulphuret of platinum is digested in nitric acid, and carefully evaporated; or when perchloride of platinum is gently heated in sulphuric acid, a dark brown solution of *persulphate of platinum* is obtained; if this solution be mixed with nitrate of baryta, sulphate of baryta is thrown down and pernitrate of platinum remains dissolved; this may be in part decomposed by solution of caustic soda, which forms a yellow precipitate, becoming brown when carefully washed and dried, and which is *hydrated peroxyd.* Heated in a retort, it first gives out water, and becomes black; at a higher temperature it evolves oxygen, and the metal is reduced; it leaves a very feeble attraction for the acids, but readily combines with many of the salifiable bases; it dissolves in the caustic and carbonated alkalies, and may be combined with lime, strontia and baryta, by adding these earths to its acid solution, when it falls in union with them, in the form of a yellow powder. (Berzelius.) It forms a fulminating ammoniacal compound, similar to fulminating gold. This oxyd consists of"

| | | | | Berzelius. | Chenevix. |
|---|---|---|---|---|---|
| Platinum, | 1 | 99 | 86 | 85-87 | 87 |
| Oxygen, | 2 | 16 | 14 | 14-13 | 13 |
| | 1 | 115 | 100 | 100-00 | 100 |

The next compound I would introduce, is that of the metal and sulphur, known as

*Sulphuret of Platinum.*—There are two of these spoken of, viz. *proto* and *bi*sulphuret of platinum—the first, (*proto-sulphuret,*) is conveniently made by exposing the two substances (sulphur and platinum) to the action of heat, in a glass tube which has been subjected to exhaustion. When the process is finished, we have a gray, pulverulent mass, which is not acted upon by the strongest acids, and which consists of one atom of platinum and one of sulphur.

*Bisulphuret of Platinum.*—With regard to this substance, Brande has the following paragraph:

"When a solution of perchloride of platinum is mixed with sulphuret of ammonia, or potassium, a black powder falls, which, when dried in vacuo, over sulphuric acid, contains, according to

Berzelius, no water. When this precipitate is exposed upon paper to dry in the air, the sulphur absorbs oxygen and becomes sulphuric acid, which chars the paper. When sulphureted hydrogen is passed through a solution of bichloride of platinum, the precipitate which falls, consists of chloride and sulphuret of platinum.

The bisulphuret consists of

| | | | | | |
|---|---|---|---|---|---|
| Platinum, . . . . . | 1 | 99 | 75-5 | 77 | 75-25 |
| Sulphur, . . . . . . | 2 | 32 | 24-5 | 23 | 24-75 |
| Bisulphuret of platinum, | 1 | 131 | 100-0 | 100 | 100-00 |

*Proto-Chloride of Platinum.*—The best method of preparing this substance, is to subject the perchloride of platinum to heat until chlorine gas no longer comes over. The resultant product is a pulverulent mass, having a gray color, and which is insoluble in the strong mineral acids and water at 60° F., though boiling hydrochloric acid will dissolve it. Red heat will decompose it, and chlorine gas will be evolved; it is also decomposed by potassa, with the elimination of the protoxyd. The constitution of this substance is, chlorine 1 + platinum 1.

"When alcohol is gradually added to a hot concentrated solution of proto-chloride of platinum, in liquid potassa, effervescence ensues from the escape of carbonic acid, and a black powder falls, which, boiled successively with small portions of alcohol, then with hydrochloric acid, and then with potassa, and lastly with repeated portions of water, is Liebig's 'platinum black;' it must be separated by decantation and dried in a porcelain capsule to avoid contact of organic matter. It is metallic platinum in a state of fine comminution; it may be heated to redness without change of appearance, but at a white heat it assumes metallic lustre; it becomes incandescent, when moistened with alcohol, oxygen is absorbed and acetic acid formed; it immediately inflames a current of hydrogen in the contact of air; it absorbs about 750 times its volume of hydrogen, and also absorbs and retains other gaseous bodies. When excess of ammonia is added to the hydrochloric solution of protochloride of platinum, and the mixture boiled, it becomes turbid, and deposits deep green acicular crystals, insoluble in water and in hydrochloric acid, which consist of 1 atom of proto-chloride of platinum, and 1 of ammonia. This salt is not affected by boiling caustic alkalies, nor by boiling sulphuric or hydrochloric acid; so that as Gros observes, it is difficult to admit that it contains 'ammonia in the state of ammonia.' When it is digested in hot nitric acid it is converted into a white granular crystalline powder, easily soluble in water, and half the platinum of the green salt is at the same time separated in the metallic state. Neither the chlorine nor the platinum of this nitric salt can be detected by the usual tests; its elements are, 1 atom of proto-chloride of platinum, 2 of ammonia, 1 of oxygen and 1 of nitric acid, so that its ultimate elements are (pla. + c + 3 n + 6 o + 6 h.) When a hot saturated solution of this nitric salt is mixed with sulphate of soda, the nitric acid is displaced by the sulphuric, and a corresponding sulphate obtained; and a hydrochlorate, phosphate, oxalate, &c., may be similarly obtained by double decomposition. These salts are represented by Gros as containing a compound base, which he compares to ammonium represented by (pla. + c + 2 n + 3 h) = B. The *nitrate* will then be (b + o + n',) the sulphate (b + o + s',) and the hydrochlorate (b + c,) &c." Vide Brande, p. 990.

*Perchloride of Platinum.*—An excellent method for the preparation of this substance is the following: Take a well made solution of the metal in nitro-muriatic acid and evaporate; after a while, the solution will assume a dark color, inclining to brownish red; very soon crystals will begin to make their appearance, and continued evaporation will result in the formation of a crystalline mass. We have now produced a substance, which is very soluble in alcohol and ether, and sparingly in water—it is the *perchloride of platinum.* The salt in question is an excellent means of ascertaining the presence of potassa; one precaution, however, being necessary to be observed in the experiment, viz. that if ammonia be present, it must be expelled by heat. The resultant, if potassa be present, is a triple salt, which will not be taken up by alcohol, and of course, will be deposited in the bottom of the vessel; if, however, potassa be not present, the solution will not even be rendered turbid.

We come next in order to describe some of the singular compounds known—*Platino-chlorides*—for the following account of which we are indebted to Brande:

"*Platino-Chlorides.*"—"Both the chlorides of platinum enter into definite combination with the chlorides of the alkaline bases, and form *platino proto-chlorides* and *platino perchlorides.*"

*Platino Proto-Chloride of Ammonia.*—(Pla. + c) + (A + h cl) is obtained by adding an equivalent of sal ammonia to 1 of proto-chloride of platinum, dissolved in hydrochloric acid, it forms deep red crystals."

"*Platino Proto-Chloride of Potassium.*—(pla + po + 2 c.) This salt was obtained by Magnus, in the form of red, anhydrous, four-sided prisms, insoluble in alcohol, by evaporating a solution of proto-chloride of platinum, and chloride of potassium, in hydrochloric acid. It consists of"

| | | | |
|---|---|---|---|
| Platinum, | 1 | 99 | 46.9 |
| Potassium, | 1 | 40 | 19.0 |
| Chlorine, | 2 | 72 | 34.1 |
| | 1 | 211 | 100.0 |

or

| | | | |
|---|---|---|---|
| Proto-chloride of platinum, | 1 | 135 | 63.9 |
| Chloride of potassium, | 1 | 76 | 36.1 |
| | 1 | 211 | 100.0 |

"*Platino Proto-Chloride of Sodium.*—Similarly obtained, is not crystallizable, and very soluble in alcohol." "Platino-bichloride of ammonia. Ammonio-muriate of platinum." (Pla. + 2 c) + (A + h cl.) This is the well known yellow powder, which falls, when solutions of perchloride of platinum and sal ammoniac are mixed. When it is exposed to heat, it loses a little water, and a compound of sal ammoniac and proto-chloride of platinum is at first formed; the ammonia is ultimately decomposed, and the platinum remains, in the peculiarly spongy state before referred to. This *ammonio-chloride* is very sparingly soluble in pure water; rather more so in water acidulated by hydrochloric acid; insoluble in alcohol. If the solution from which it is precipitated contain *irridium* or *palladium,* it has a tawny-red color; these may be removed by boiling in dilute nitric acid, and filtering the red solution, whilst hot; as it cools, it deposits a red crystalline powder, which is generally a triple salt of *irridium*, and, from which, the acid may be poured off for use as before. This double chloride, when carefully dried, contains between 44 and 45 per cent. of platinum; it is composed of"

| | | | | | | | |
|---|---|---|---|---|---|---|---|
| Platinum, | 1 | 99 | 43-2 | Bichloride of platinum, | 1 | 171 | 75-9 |
| Chlorine, | 2 | 72 | 32-4 | | | | |
| Ammonia, | 1 | 17 | 7-7 | Hydro-chlorate of ammonia, | 1 | 54 | 24-1 |
| Hydro-chloric acid, | 1 | 37 | 16-7 | | | | |
| Platino-bichloride of ammonia, | 1 | 225 | 100-0 | | 1 | 225 | 100-0 |

"*Platino-Bichloride of Potassium.*—(Pla. + 2 c) + (po + c).—This salt is thrown down in the form of a yellow powder, when solutions of chloride of potassium and of bichloride of platinum are mixed; it is very sparingly soluble in water, and is deposited from its boiling solution in small octohedral crystals; it is insoluble in alcohol; when heated, it evolves chlorine, and leaves a mixture of metallic platinum and chloride of potassium. Its difficult solubility, renders bichloride of platinum, as already stated, a useful test of the presence of the salts of potassa. It is anhydrous, and contains"

| | | | |
|---|---|---|---|
| Bichloride of platinum, | 1 | 171 | 69-5 |
| Chloride of potassium, | 1 | 76 | 30-5 |
| Platino-bichloride of potassium, | 1 | 247 | 100-0 |

"*Platino-Bichloride of Sodium.*—(Pla + 2 c)+(so + c.)—Chloride of sodium occasions no precipitate with bichloride of platinum, but the mixed solutions yield on evaporation prismatic or tabular crystals, of a deep orange color, soluble in water and alcohol, and which, when heated, lose 19-25 per cent. of water of crystallization, and leave the anhydrous double salt; the crystals, therefore, contain"

| | | | |
|---|---|---|---|
| Bichloride of platinum, . . | 1 | 171 | 60-0. |
| Chloride of sodium, . . . | 1 | 60 | 20-8. |
| Water, . . . . | 6 | 54 | 19-2. |
| Crystals of platino-bichloride of sodium, | 1 | 285 | 100-0- |

"*Platino-Bichloride of Barium.*—When baryta-water is gradually added to a solution of bichloride of platinum, a precipitate falls, composed of baryta and peroxyd of platinum, (platinate of baryta.) The solution contains excess of baryta, which falls in the shape of carbonate by exposure to air, and afterwards small crystals of the double salt are deposited. This salt may also be formed by mixing the two chlorides in atomic proportions: the crystals are orange-colored, and in form and appearance, resemble those of chromate of lead. They consist of

| | | | |
|---|---|---|---|
| Bichloride of platinum, . . | 1 | 171 | 54-56. |
| Chloride of barium, . . | 1 | 105 | 33-75. |
| Water, . . . . | 4 | 36 | 11-69. |
| | — | —— | ——— |
| Crystals of platino-chloride of barium, | 1 | 312 | 100-00." |

"Platino-chloride of *calcium* and *strontium* have been described by Bonsdorff, as also those of *magnesium*, *manganese*, *iron*, *zinc*, *cadmium*, *copper*, *nickel* and *cobalt*; the crystals of the last eight, are *isomorphous*, and consist of 1 atom of the bichloride of platinum, 1 atom of the basic chloride, and 6 atoms of water."

"Platino-chloride of silver, is thrown down as a yellow basic salt, when solutions of bichloride of platinum and nitrate of silver are mixed; the residuary liquid remains colorless: boiling hydrochloric acid, abstracts the chloride of platinum, and leaves the chloride of silver, nearly colorless." (Vanquelin.)

*Phosphuret of Platinum.*—For the preparation of this compound, it is necessary to expose the metal to the action of heat, in union with clear phosphorus, (the clippings of the metal are most economical,) when we have as a resultant—*phosphuret of platinum*, the properties of which are but imperfectly known; it consists of platinum 1—phosphorus 1.

*Perphosphuret of Platinum*, is made by a process similar to the above, using *ammonia chloride of platinum* instead of the metal, and applying heat. Its exact constitution is uncertain.

*Ferro-cyanuret of Platinum.*—To obtain this substance, an excellent method is to take the spongy platinum, 1 part—and ferro-cyanuret of potassium, 1 part—and expose them to a heat below redness; decomposition takes place, attended with the elimination of iron, which comes from the cyanogen. Having progressed thus far, dissolve the product in water, and then by careful management, crystals may be obtained, which are bi-colored if viewed in different positions, changing from blue to yellow. The salt is rather insoluble in cold water, but will dissolve with some freedom in water at 212° F., it cannot, however, be preserved in this menstruum, unless the temperature be kept up; as the solution cools, crystals will be deposited, but we have not yet obtained absolutely positive results.

*Sulphate of Platinum.*—To obtain this salt, first make a solution of the oxyd of platinum in potassa, and add to it sulphuric acid; a precipitate is formed, which, when the liquid is decanted, may be dissolved in sulphuric acid which has been well diluted: evaporate now, and the result is very dark colored, becoming red on the addition of water. From the solution of sulphate of platinum, caustic potash will throw down the oxyd of the metal. *Persulphate of platinum*, considered an excellent test for gelatine, is obtained by adding nitric acid, to the sulphurets.

*Nitrate of Platinum*, is formed by adding nitric acid to the protoxide of platinum, and the pernitrate is formed by adding the same acid to the peroxyd.

The following account of "fulminating platinum and the characters of the salts of platinum," is from Brande.

"*Fulminating Platinum.*—E. Davy says, that the precipitate from solution of sulphate of platinum by a slight excess of ammonia, when boiled in potassa—washed and dried, was a *fulminating platinum:* it explodes at 420° with a loud report, and appears to be a compound of oxyd of platinum, ammonia and water. (Phil. Trans., 1817.) He has also described a compound of platinum, (Phil. Trans., 1820, p. 108,) obtained by mixing equal volumes of strong aqueous solution of the sulphate and of alcohol. The color of the sulphate slowly disappears, and in some days, a black substance subsides, which is washed and dried. It is also formed by boiling the sulphate and alcohol together for a few minutes. This substance is permanent in the air, and insoluble in water. It detonates feebly when heated, and is not affected by chlorine, nor by nitric, sulphuric or phosphoric acids; but it is slowly soluble in hydrochloric acid. Put into liquid ammonia, it acquires fulminating properties; and plunged into ammoniacal gas, it becomes red-hot: the same phenomenon is exhibited by exposing it to the vapor of alcohol, or by placing it upon a piece of paper moistened with that fluid: in these cases, the platinum is reduced, with the evolution of heat, and the ignition seems to depend upon the slow combustion of the vapor of the alcohol. Some of these properties correspond with those of *platinum black.*"

*Characters of the Salts of Platinum.*—The difficult solubility of the ammonio and potassio-chlorides of platinum, and the solubility of the soda-compounds, are very characteristic of this metal. Phosphate of soda, produces no precipitate in chloride of platinum; the ferro-cyanuret of potassium, throws down the platino-chloride of potassium: cyanuret of mercury occasions no precipitate: iodide of potassium communicates a reddish-brown color, to solutions of the chlorides of platinum, and gradually produces a brown precipitate; and if the mixture be heated in a matrass, the glass acquires a coating of metallic platinum, but its complete separation in the metallic state is slow; iron, zinc, cadmium and copper, are its most effective precipitants; they separate it as a black powder, which sometimes adheres in fibres to the glass."

The subject of the next article will be Mercury.

[To be continued.]

